# 83 Barriers and Enablers to Interrupting Sedentary Behaviour when Working from Home in a Desk-Based Occupation: A Qualitative Exploration of the Older Employee Experience

**DOI:** 10.1093/eurpub/ckae114.151

**Published:** 2024-09-26

**Authors:** Lily Mott, Annemarie Money, Amelia Parchment, Sheena Johnson, Chris Todd

**Affiliations:** The University Of Manchester, United Kingdom; The University Of Manchester, United Kingdom; The University Of Manchester, United Kingdom; Alliance Manchester Business School, United Kingdom; The University Of Manchester, United Kingdom

## Abstract

**Purpose:**

Those employed in desk-based occupations sit for large amounts of their working day, often in long uninterrupted bouts, exposing them to the health risks associated with sedentary behaviour (SB). With more workplaces offering home-working options, it is important to explore how this environment influences SB. With the number of older workers (≥50) increasing, it is vital to promote healthy ageing in the workplace and ensure this population are not put at increased risk due to occupational sitting. This is especially important considering the increased risk of health conditions as we age. This study aims to use the Theoretical Domains Framework (TDF) alongside the Capability Opportunity Motivation Behaviour (COM-B) model to identify the barriers and enablers to interrupting and reducing SB in older employees (≥50) working from home in desk-based occupations. Targeting older employees in the homeworking environment is an understudied area of research.

**Methods:**

Twenty-two participants aged ≥50 took part in one-to-one semi-structured Zoom interviews from an employer within Greater Manchester. Reflexive thematic analysis is being used to inductively develop themes and sub-themes which will be then deductively mapped and linked to the COM-B and TDF.

**Results:**

Initial responses from participants reveal workload and the nature of work tasks as a key barrier to interrupting SB when working from home. Participants shared they often forget to break up their sitting, demonstrating the habitual nature of SB. Final interviews are being conducted with data analysis to be completed in time for the conference where a more specific presentation of main themes and sub-themes will be presented.

**Conclusions:**

As working from home is imbedded in many workplaces, it is critical to develop a better understanding of how this setting may influence SB in desk-based older workers. This will help inform the development of suitable interventions to reduce and interrupt SB to promote healthy ageing at work prior to retirement.

**Support/Funding Source:**

Dunhill Medical Trust, The NIHR, The University of Manchester

